# Weight-supported training of the upper extremity in children with cerebral palsy: a motor learning study

**DOI:** 10.1186/s12984-017-0293-3

**Published:** 2017-08-30

**Authors:** Jeffrey W. Keller, Hubertus J.A. van Hedel

**Affiliations:** 1 Rehabilitation Center Affoltern am Albis, University Children’s Hospital Zurich, Mühlebergstrasse 104, -8910 Affoltern am Albis, Switzerland; 2 Children’s Research Center, University Children’s Hospital Zurich, Zurich, Switzerland; 3 Department of Human Movement Sciences and Sports, ETH Zurich, Zurich, Switzerland; 4 Doctoral Program Clinical Science, Faculty of Medicine, University of Zurich, Zurich, Switzerland

**Keywords:** Armeo spring, Skill acquisition, Transfer, Retention, Neurorehabilitation, Exergame, Pediatric, Adolescent, Congenital brain lesion

## Abstract

**Background:**

Novel neurorehabilitation technologies build upon treatment principles derived from motor learning studies. However, few studies have investigated motor learning with assistive devices in children and adolescents with Cerebral Palsy (CP). The aim of this study was to investigate whether children with CP who trained with weight support in a playful, virtual environment would improve upper extremity task performance (i.e. skill acquisition), transfer, and retention, three aspects that indicate whether motor learning might have occurred or not.

**Methods:**

Eleven children with CP (mean age 13.3 years, standard deviation 3.4 years), who were mildly to moderately impaired, participated. They played in the Armeo® Spring the exergame Moorhuhn with their more affected arm during 3 days (70 min pure play time). For this within-subject design, kinematic assessments, the Box and Block Test, and five items of the Melbourne Assessment were administered twice during a baseline week (one week before the intervention), directly before and after the intervention, and one day after the training phase (retention).

**Results:**

The average exergame score improved from 209.55 to 339.73 (*p* < 0.001, Cohen’s d = 1.80), indicating skill acquisition. The change in the Box and Block test improved from 0.45 (baseline week) to 3.95 (intervention week; *p* = 0.008, d = 1.59) indicating skill transfer. The kinematic assessments and the Melbourne items did not change. Improvement in game score and Box and Bock Test persisted one day later (retention).

**Conclusions:**

We found evidence indicating the successful acquisition, transfer, and retention of upper extremity skills in children with CP. We therefore infer that motor learning occurred when children with CP trained their more affected arm with weight-support in a playful, virtual environment.

## Background

Cerebral palsy (CP) is an umbrella term for a group of chronic disorders caused by nonprogressive cerebral abnormalities which occur before birth or early in life and lead to motor impairments and thus, activity limitations [1, 2]. CP affects 2 to 3 children out of 1000 live births [3]. In addition to motor control deficits, children with CP often have comorbidities such as reduced sensibility, cognition, tonus, and strength. These deficits can lead to impairments in daily living ranging from barely noticeable to very limiting [2, 4]. Consequently, one major goal in the habilitation process of children affected by CP is to improve motor control and thereby increase independence, participation, and overall quality of life. Oftentimes, this includes intensive training of the upper extremities, performed under the guidance of occupational therapists [5].

In addition to conventional occupational therapy, robots and weight-supporting systems might be beneficial for improving upper limb skills. The systems allow performing an increased number of repetitions per session and the exergames provide enhanced feedback as well as motivational components [6, 7]. These factors are crucial for a successful therapy in general and particularly in pediatric rehabilitation.

Unfortunately, for children with CP, the effectiveness of such applications has rarely been studied. A recent systematic review [8] evaluated the effectiveness of upper limb robotics devices in children with CP. Of the nine studies that they identified, seven were case studies. In these studies, three systems were used, the InMotion2 [9, 10], the NJIT-RAVR [11], and CosmoBot [12]. The authors of the review confirmed the potential for robotic therapy to improve upper limb function in children with CP. However, even when considering the positive results of the randomized controlled trial from Gilliaux et al. [13], who investigated the effectiveness of the REAPlan robot in children with CP, we agree with the authors of the review that the paucity of group design studies summoned the need for more rigorous research before conclusive recommendations can be made [8]. While more rigorous randomized controlled trials are needed to establish the evidence, we think there are still many open questions that would improve our understanding of applying such systems and could improve the design of large-scaled studies and the selection of appropriate outcome measures. Examples of such questions are: which body functions specifically improve during an exergame training with a particular device, how well can improvements be transferred to other, clinically more relevant, functions or activities, and how long can the achieved improvements be retained?

These questions relate directly to the field of motor learning. Motor learning includes motor adaptation, decision-making, and skill acquisition [6, 14]. Krakauer [6] characterizes skill acquisition as *“practice-dependent reduction of kinematic (geometry and speed of movement) and dynamic (forces necessary to generate movement) performance errors”*. This means that over time the practiced movement will become smoother, faster, more accurate, and more efficient. These factors contribute to an improved performance and thus indicate the successful acquisition of a skill. However, motor learning also implies the retention and transfer of a movement, both of which are of crucial importance for a (re)habilitation process [6, 9]. If a patient practices reaching for a cup and shows improvements during the training session (acquisition) this progress should, to some extent, still be present at a later time point (retention). Furthermore, to be useful in daily life, the movement should be transferable (or generalizable) to different settings, such as a different environment (e.g. grasping the cup from the table or the higher cupboard) or grasping a different cup. Despite the significance of motor learning for neurorehabilitation, very few studies have evaluated the influence of neurological conditions on motor learning processes [14]. This is especially true for research in the habilitation process of children affected by CP in combination with novel technologies for upper extremity therapy (see, for some examples [9, 13]).

**Fig. 1:**
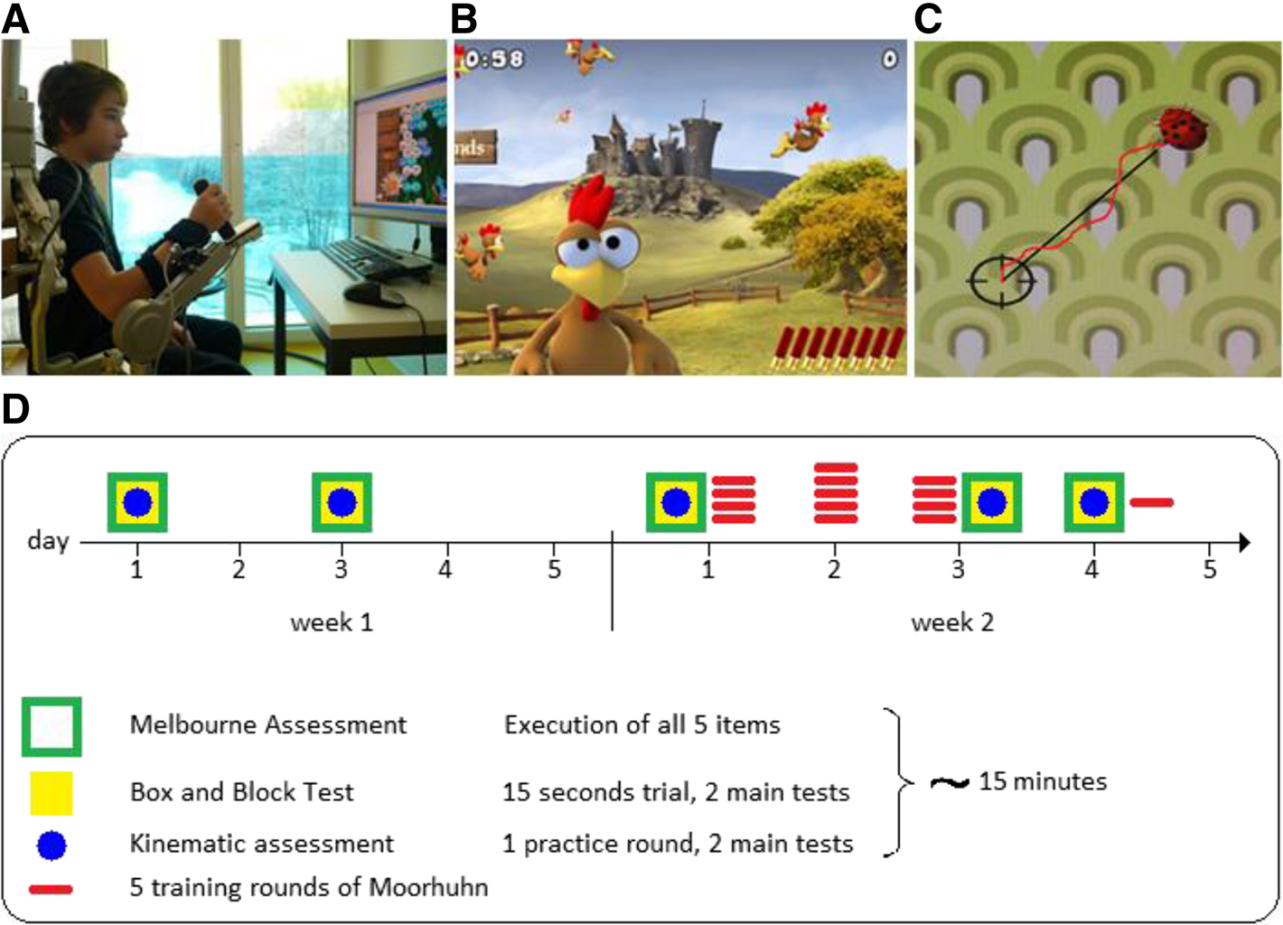
**a** Picture showing a participant training with the ARMEO® Spring. **b** Screen shot of the game Moorhuhn (“Crazy Chicken”) where the goal is to get as many points as possible by shooting the birds. Hitting smaller targets was rewarded with receiving more points. **c** Screen shot of the kinematic assessment “vertical catch 2D”. The parameters recorded are time to catch all 12 targets and path-ratio. Dividing the performed path (marked in red) by the ideal path (straight line between target and crosshairs in black) yields the dimensionless quantity path-ratio, which can at best reach a value of 1. **d** Depicts the procedure time line. Week 1 contains the baseline measurements and week 2 the measurements before and after the intervention. By comparing the differences between both weeks, this within-subject design enabled us to account for various factors such as the effect of other therapies, time of day, and repetition of the assessments

Therefore, the aim of this study was to assess whether training with weight support in a playful, virtual environment could result in an acquisition, transfer, and retention of upper limb skills in children with CP. We hypothesized that training with the ARMEO® Spring (Hocoma AG, Volketswil, Switzerland, see Fig. 1a), a weight-supporting device developed for training the upper extremity, would lead to an increased exergame performance. Furthermore, we hypothesized that the improvements would translate to kinematic parameters of other tasks and clinically more relevant tests and that these improvements are retained up to 24 h after finishing the trials. While we originally strived for a longer retention period (e.g. one week later), we decided to reduce this period to 1 day, because the average length of stay of patients in our rehabilitation center certainly would have reduced the number of participants. For those, who were still inpatients one week after the 1-day retention assessment, we also performed an 8-day retention assessment. As conventional therapies took place also during the intervention phase, we monitored these therapies to account for them.

## Methods

### Participants

We recruited in-patients from the Rehabilitation Centre Affoltern am Albis and the Stiftung Vivendra in Dielsdorf, Switzerland, from September 2014 to March 2015. The goal was to recruit between 10 and 15 participants, which would be in line with the number of patients that participated in previous studies [9, 13]. The inclusion criteria were diagnosis of CP, age between 6 and 18 years (the exergame was approved for 6 years and older), ability to understand and follow simple instructions, ability to sit upright for 45 min, and ability to play the exergame Moorhuhn (Fig. 1b) with the ARMEO® Spring. Furthermore, they had to have a Manual Ability Classification System (MACS) level of III or less. The MACS classifies how children with CP handle objects in daily activities [15]. It assesses the collaboration of both hands and allocates the performance to one of five categories. Children at level I handle objects easily and successfully whereas children at level V do not handle objects and have severely limited ability to perform even simple actions. Level III refers to the ability to manipulate objects in a prearranged setting without assistance.

Exclusion criteria were according to the guidelines of the ARMEO® Spring (e.g. skin lesions, visual deficits, visually evoked seizures; for further information see the guidelines). Additionally, patients with surgical interventions or Botulinum toxin treatment on the upper extremities in the past 6 or 3 months, respectively, were excluded.

To describe the participants, we assessed the following characteristics: age and gender, CP category, more affected arm, dominant arm, and MACS level. Furthermore, we assessed the Functional Independence Measure for children (WeeFIM) and the Gross Motor Function Classification System (GMFCS). The WeeFIM is a tool that rates the functional independence of children in everyday life situations using a 7-level ordinal scale. A score of 7 indicates total independence, a score of 1 that the subject needs total assistance. The assessment consists of three main domains: self-care, mobility, and cognition with a total of 18 items (minimal score 18, maximal 126) [16]. The GMFCS consists of 5 levels and was developed to standardize the classification of the gross motor function, with emphasis on trunk control and walking of children with CP [17]. Children at level I can perform all the activities normally developing children of the same age can, except there may be some limitation in speed and quality of movement. Children at level V display difficulties in head and trunk control in most positions or achieving any voluntary control of movement at all [18].

### Study design

The study lasted 2 weeks for each participant. During week 1, the baseline week, several kinematic (Fig. 1c and d) and clinical assessments were performed, approximately 48 h in between, to provide us an insight into how the other therapies, the repetition of the assessments, the time of day, and other factors influenced the outcome measures.

During week 2, the participants performed the same assessments at the same time interval, but additionally in-between the measurements, the children participated in training sessions with the ARMEO® Spring and the exergame Moorhuhn. In total, the participants played 70 trials (each trial lasted 1 min) of Moorhuhn during these 3 days. The distribution of playtime across the 3 intervention days was as follows: day 1: 4 blocks × 5 trials (total: 20 trials), day 2: 6 blocks × 5 trials (total: 30 trials), and day 3: 4 blocks × 5 trials (total: 20 trials). During the 3 days of exergaming, we could determine *skill acquisition* by monitoring changes in the game scores. *Skill transfer* was quantified by evaluating the changes in the kinematic and clinical assessments, and we could adjust for potential changes due to the regular conventional therapies using the measurements of week 1. About 24 h after the last training, all assessments and 5 trials of the exergame Moorhuhn were carried out again. This enabled us to determine the *retention*. If possible, we repeated the assessments one week later. The exergame intervention was performed with the participant’s more affected arm. All assessments and interventions had to fit within the participant’s daily schedule and therefore lasted maximally 45 min.

### Device: Pediatric ARMEO® spring

The ARMEO® Spring is a passive system offering weight support through an adjustable spring mechanism (Fig. 1b). The ARMEO® Spring is initially based on the T-WREX [19] and was then commercialized by the company. Our center received in 2010 the pediatric device and the occupational therapists have worked with it since. For all measurements, we used the ‘Armeocontrol 1.24’ software version.

The ARMEO® Spring (Fig. 1a) allows the patient to perform self-initiated movements within a 3 dimensional workspace, enhancing any residual function. Therapeutic goals are to improve or maintain reach, grasp and transfer movements, active range of motion, force regulation and initiation of movement [20]. The adaptable exoskeleton is attached to the patient’s arm at the upper and lower arm and near the wrist. The position sensors and software enable training in a virtual environment with augmented feedback. Using a screw, the tension of two springs, one for the upper arm and one for the lower, can be adjusted to increase the weight support. As it is difficult to quantify the exact amount of weight support for each position of the arm in 3-dimensional space, the amount of weight support is numbered for the upper arm from A (almost no tension, i.e. minimal weight support) to I (maximal tension, i.e. maximal weight support) and for the lower arm from A to E. To our knowledge, research on the application of the ARMEO® Spring in children with CP has rarely been performed. We found a single case study [21], a conference paper describing the applicability in children with CP [22] and one study in which the authors developed a measure that should automatically quantify the performance of patients during upper limb robotic training [23].

### Intervention: The Moorhuhn exergame

Moorhuhn (Fig. 1b), in English speaking countries distributed as “Crazy Chicken”, is an exergame with the goal of hunting birds of various sizes (and thereby various points). It requires the player to move and position the arm and hand quickly and accurately in the virtual environment and timely grasp the joystick to shoot at a chicken. It is possible to pivot within the game. This means that by moving the cursor (i.e. moving the arm) to the far right or left of the game the location within the virtual environment can be shifted to search for newly appeared birds. The workspace of this exergame was adjusted for each patient individually before the first training round of each day. To determine the workspace, patients positioned in the ARMEO® Spring had to lift the arm up and down, move as far as possible to the right and left, and point forward and towards the chest. The Moorhuhn game was calibrated according to these settings. This ensured that the participants had to use their maximum movement amplitude to pivot the screen and reach all possible targets, making training sessions as challenging as possible. In addition to training shoulder, elbow, and grasping movements, the game also requires pronation and supination of the forearm after 8 shots for reloading.

On the first intervention day (day 1 of week 2, see Fig. 1d), there was a demonstration of the game and a one minute trial session that was not included in the data analysis. The reason for this was to explain the game with all its controls, hidden points, and the pivoting, since these may be sources of bias influencing the learning curve. By introducing these elements before the training phase, we hoped to reveal the actual performance gains.

In the ARMEO® Spring version of this game the moving targets are large, medium, and small birds and account for 5, 10, and 25 points, respectively. Additional points can be earned/lost by hitting “special targets”: There are giant birds (+25 points) appearing randomly with an acoustic cue and vanishing after a few seconds, 4 chickens attached to a windmill (+25 points each), the top hat of a scarecrow (+25 points), the signpost (−25 points for each hit), and the Hocoma-airplanes (−25 points). All targets, except for the “special ones”, moved throughout the entire trial. The total game score was documented for all training rounds.

The difficulty setting of the exergame has an influence on duration of the training round and target speed. To standardize the protocol, we set the game to ‘very easy’ for all participants and all training days. This setting also enabled participants with more severe impairments to play the game. Playing at this difficulty level meant that each round lasted 1 min and the main targets moved at the slowest possible speed. This difficulty level also ensured that the presentation of the ‘special targets’ (top hat, windmill chickens) was comparable between different rounds. Differences in game scores per round might, therefore, reflect changes in game performance of the patient rather than differences in the appearance of targets.

The difficulty level was independent of the amount of weight support of the arm. At onset, the investigator determined the level of weight support of the upper and lower arm. Criteria were that the weight of the ARMEO® Spring was compensated for and that the patient reported that he or she could lift the arm without the weight of the exoskeleton pulling them down. If it was hard to lift the arm, the springs were adjusted until the patient reported that lifting was possible. We noted the upper and lower arm lengths and spring settings, which allowed the same settings and weight support for each patient on each occasion.

### Kinematic and clinical assessments

The assessments (represented in green, yellow, and blue in Fig. 1d) consisted of a kinematic assessment (Fig. 1c), the Box and Block Test (BBT), and the Melbourne Assessment of Unilateral Upper Limb Function (Melbourne Assessment). Participants first performed the 5 items of the Melbourne Assessment, then the BBT, and finally, the kinematic assessments in the ARMEO® Spring.

#### Kinematic assessment (vertical catch 2D)

The goal of this game-like assessment is to catch ladybugs that appear on the screen (Fig. 1c). We used only the ‘very easy’ level, which means that the participant had to direct the hair cross on the appearing ladybug as quickly as possible. The onset of each consecutive movement was the position of the previously caught ladybug. During one assessment, 12 ladybugs had to be caught. While the position of the 12 ladybugs was scattered over the screen, the order and position were the same for each assessment (i.e. that, for example, the ladybug in the far right corner always appeared last). The kinematic outcomes were average path-ratio (dimensionless quantity; calculated as performed path divided by the shortest possible one) and total time (measured in seconds) needed to catch all 12 targets.

As opposed to the Moorhuhn exergame, the targets do not move and the workspace is predefined, meaning it cannot be adjusted. While this makes it more challenging for patients with less range of motion, it improves the standardization and makes the results comparable.

Prior to each actual testing, the participant got one practice round to be able to adjust to the new setting and so the ARMEO® Spring could be repositioned, if necessary. The participant performed then this assessment twice (i.e. in total 24 ladybugs) and the results were averaged.

#### Box and block Test

The BBT is a measure of unilateral gross manual dexterity. The participants sat on a chair, feet on the ground, facing a table with a two-compartment box on it. The side to be tested contained 150 wooden cubes with an edge length of 2.5 cm each. The goal of this assessment is to move as many blocks as possible from one side to the other in 60 s. In their study, Mathiowetz et al. [24] provided detailed standardized instructions. In this study, prior to the main trials, participants performed a 15 s practice trial [25]. Then, they performed the BBT with the more affected arm twice. We averaged the results.

#### Melbourne assessment of unilateral upper limb function

The Melbourne Assessment measures the quality of unilateral upper limb function in children with neurological impairments [26]. The test is composed of 16 items and assesses the quality of movement. For the items that we chose, up to 4 criteria were evaluated: (1) active range of motion, (2) accuracy, (3) dexterity of finger movement, and (4) smoothness of the movement [27]. For this study, we used the 5 items we expected to show the most changes, because parts of them were trained intensively by playing Moorhuhn. Item 3: Reach sideways to an elevated position (max. Score: 9). Item 4: Grasp of crayon (max. Score: 4). Item 6: Release of crayon (max. Score: 10). Item 10: Pointing (max. Score: 16), and Item 15: Reach to opposite shoulder (max. Score: 9).

Every item was filmed and scored according to the prescribed criteria. The total points possible for this setup were 48. As outcome measure, we looked at the percentage score, so the achieved score divided by the maximum total score multiplied by 100%.

### Possible confounder: Number of therapies between assessments

All measurements and training sessions were performed in addition to the daily rehabilitation routine. Therefore, we monitored the number and type of therapies between the first and second assessment of each week. We considered the following therapies relevant: occupational therapy, robotic therapy for the upper extremity, physiotherapy, medical training therapy, particular sports groups (e.g. wheelchair or coordination groups, basketball or other games), handicrafts, and other activities involving hand functions (e.g. gardening).

### Data analysis

#### Skill acquisition

*Game score:* We graphed a learning curve of the game score by averaging the data for all participants for each trial. For the statistical analyses, we averaged the scores of 5 trials of all patients to one block. To test whether the participants significantly improved their game scores, we compared block 2 of day 1 to the last block of day 3 (block 14). We decided to use block 2 (and not block 1) to omit the familiarization phase where participants got used to the device, the controls, and the surroundings of the game.

#### Transfer

We investigated the transfer (or generalization) by determining differences in the kinematic parameters (path-ratio and time needed), the BBT, and the Melbourne. To adjust for the influence of conventional therapies on the changes of these kinematic and clinical outcome measures, we compared the difference of week 1 (day 1 subtracted from day 3) with the difference of week 2.

#### Retention

Finally, we tested the retention of the game score, the kinematic measures, the BBT, and the Melbourne items. We compared the measures at one day after the final training to those achieved at the final day of the intervention. For the game scores this meant that block 15 was compared to block 14. For the kinematic measures, the BBT, and the Melbourne, we compared the measurements made on day 4 of week 2 to those achieved on day 3 of week 2. We also analyzed the 8-day retention data we were able to collect by comparing day 3 of week 2 with day 4 of week 3.

### Statistical analysis

The statistical analyses were performed with SPSS 20.0 (SPSS Inc., Chicago IL, USA). The significance level was set at α = 0.05 for all tests. We provided means and standard deviations in tabular form (to allow sample size calculations for future studies) and used box and whisker plots to illustrate the characteristics of the data.

We used the Shapiro-Wilk test to analyze the distribution of our data. Based on whether the data were normally distributed or not, we selected the corresponding parametric (paired t-test) or non-parametric test (Wilcoxon signed rank test). Since the data of the Melbourne Assessment is measured on an ordinal scale, we always used the non-parametric test. The game scores of block 14 were used in 2 analyses: to investigate skill acquisition (block 2 versus 14) and retention (block 14 versus 15). To account for the inflated error rates associated with these multiple comparisons, we applied a Bonferroni correction.

In addition to providing the test statistic and the significance score for all comparisons made, we also noted the effect size to indicate how meaningful differences were. When applying a parametric test we calculated Cohen’s d, where d exceeding 0.2, 0.5, or 0.8 represented a small, medium, and large effect size, respectively. When using a non-parametric test we calculated r_ES_, where r_ES_ exceeding 0.1, 0.3, or 0.5 represented a small, medium, and large effect size, respectively [28]. The effect sizes were calculated as follows:

$$ d=\frac{{\mathrm{mean}}_1-{\mathrm{mean}}_2}{\sqrt{\left({{\mathrm{SD}}_1}^2+{{\mathrm{SD}}_2}^2\right)/2}}\kern6em {\mathrm{r}}_{\mathrm{ES}}=\frac{\mathrm{z}\hbox{-} \mathrm{score}}{\sqrt{\#\mathrm{Observations}}} $$

Where SD stands for standard deviation and the z-score is divided by the square root of the number of total observations made.

We compared the number of therapy sessions between the first and second assessment for each week. If the paired t-test or a Wilcoxon signed rank test resulted in a significant difference between the weeks, we calculated parametric (Pearson’s r) or non-parametric (Spearman’s r_s_) correlation coefficients between the differences in number of therapy sessions (week 2 – week 1) versus the differences in outcome measures (week 2, day 3 – day 1). Correlation coefficients were interpreted as small, medium or large when r/r_s_ exceeded 0.1, 0.3 or 0.5, respectively [29].

## Results

### Participants

We had to exclude 2 from 13 participants due to lack of compliance (ID4 and ID5, see Table 1). The other participants completed all measurements of the first two weeks. The 2 girls and 9 boys that completed the study had a mean age of 13.3 years with a standard deviation (SD) of 3.4 years. For 7 children the more affected arm was the right, for the other 4 the left one. The median MACS and GMFCS levels were 2. The median total WeeFIM score was 105. Table 1 presents also the ARMEO® Spring upper and lower arm length and spring tension settings.

**Table 1:** Patients’ characteristics and device settings

ID	Age [y]	Sex	Cerebral Palsy categories	More affected arm	Dominant arm	MACS	GMFCS	WeeFIM				Arm length and spring settings	
								Total	S-C	Mob	Cog	Upper	Lower
1*	14.7	m	Mixed: dystonia and ataxic	Right	Left	2	1	98	47	18	33	9 / E	7 / A
2*	14.4	m	Mixed: bilateral spastic and ataxic	Left	Right	2	2	114	50	35	29	3 / E	11 / C
3	8.3	m	Bilateral spastic	Right	Left	2	3	85	36	19	30	0 / C	3 / A + ½
4	9.1	m	Bilateral spastic	Left	Right	1	3	95	44	30	21	6 / B	6 / A
5	12	m	Unilateral spastic	Left	Right	2	2	48	26	5	17	6 / E	6 / C
6*	9.3	f	Bilateral spastic	Left	Right	1	2	110	51	31	28	0 / C	6 / B
7*	13.7	m	Mixed: ataxic, dystonia and bilateral spastic	Right	Left	2	2	100	49	28	23	6 / D	10 / A
8*	14.6	f	Ataxic	Left	Right	2	2	94	44	16	34	5 / D	9 / A
9	17.9	m	Unilateral spastic	Right	Left	3	2	105	46	33	26	12 / F	12 / C
10	18	m	Ataxic	Left	Right	2	1	124	54	35	35	2 / E	9 / A
11	13.3	m	Unilateral spastic	Right	Left	3	1	111	47	30	34	0 / D	4 / A
12	8.4	m	Unilateral spastic	Right	Left	2	1	122	56	31	35	0 / D	5 / A
13*	13.4	m	Mixed: dystonia and bilateral spastic	Right	Left	2	4	75	38	18	19	0 / D	6 / A

Patients with ID 4 and 5 had to be excluded due to compliance issues. Patients marked with a superscript star (*) additionally performed the 8-day-retention test. The length and spring (for the weight support) settings of the upper and lower arm of the ARMEO Spring® could vary between 0 and 12 (upper arm length), 1–12 (lower arm length), A-I (upper arm spring tension), and A-E (lower arm spring tension)

*Abbreviations*: *ID* identification number, *f* female, *m* male, *MACS* Manual Ability Classification System, *GMFCS* Gross Motor Function Classification System, *WeeFIM* pediatric Functional Independence Measure, *S-C* self-care domain, *Mob* mobility domain, *Cog* cognition domain

### Skill acquisition

**Fig. 2:**
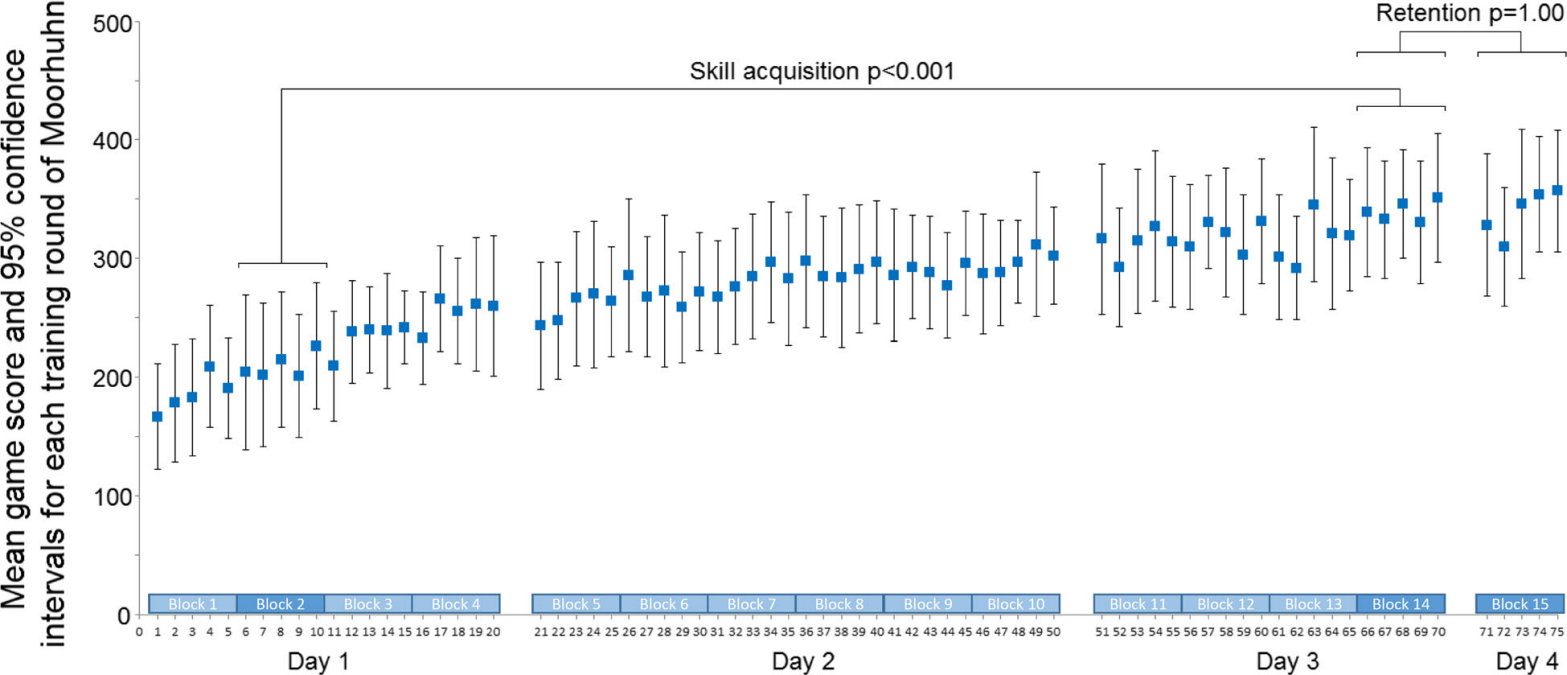
Learning curve with 95% confidence interval of the average game score showing the acquisition of the game. The segregations represent the 3 consecutive training days (day 1: trials 1–20; day 2: trials 21–50; day 3: trials 51–70) and the retention day 4 (trials 71–75). The confidence intervals were calculated as follows: 95%CI = mean ± t-value*SEM, where t-value = 2.228 (DF = 10) and SEM = SD/√n. The following power function approximated the mean data-points best: y = 148.37 × ^0.1847^, R^2^ = 93%. Abbreviations: CI, confidence interval; DF, degrees of freedom; SEM, standard error of the mean; SD, standard deviation; R^2^, explained variance

The game scores of the 11 participants resulted in a learning curve (Fig. 2). A paired-t-test showed a significant improvement [*t*(10) = 11.00, *p* < 0.001, Bonferroni corrected] in game score between block 2 (mean = 209.55, SD = 78.34) and block 14 (mean = 339.73, SD = 66.08). The effect size was large (d = 1.80).

### Transfer

**Fig. 3:**
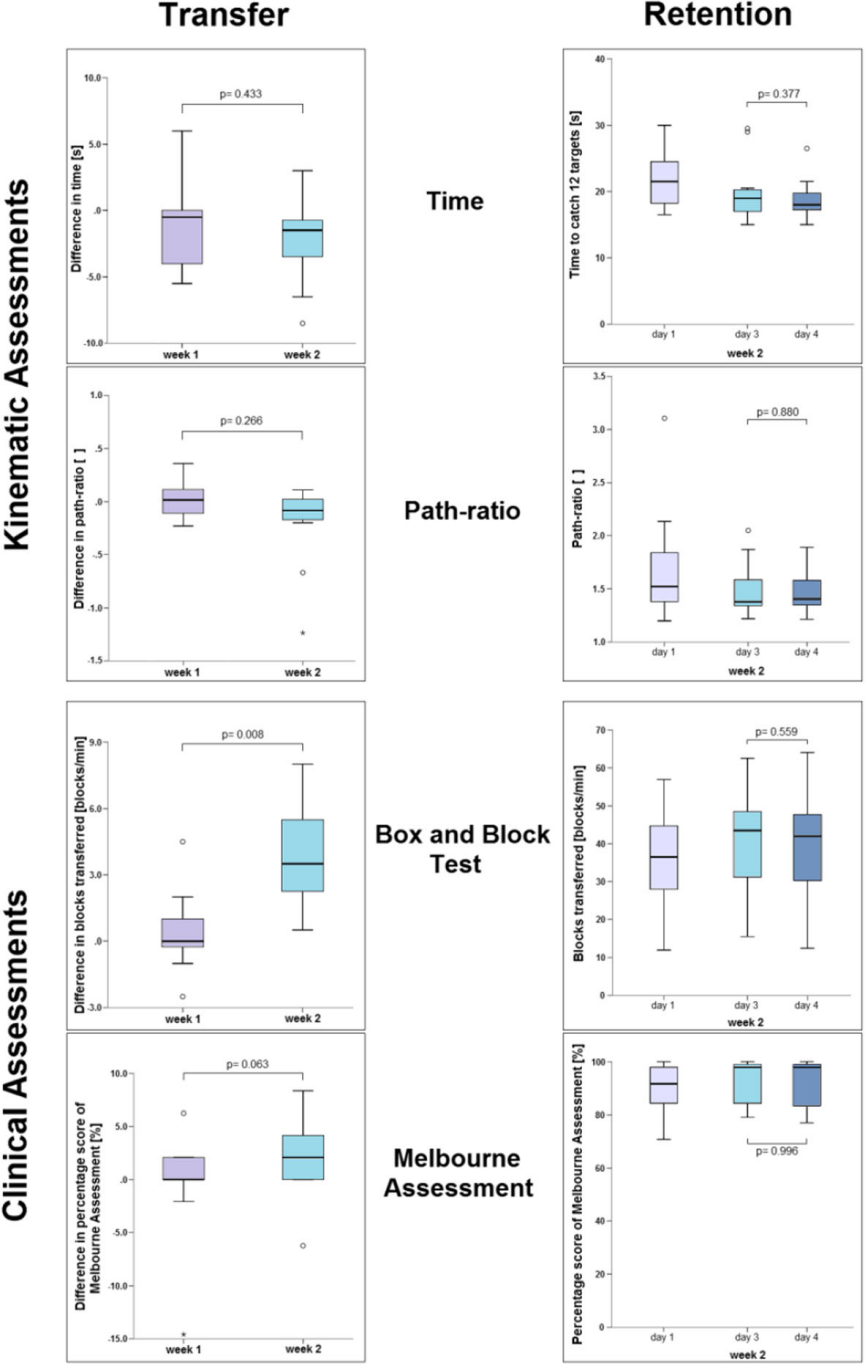
Box and whisker plots of the kinematic and clinical assessments. Circles mark outliers that rest between 1.5- and 3-times the inter quartile range, stars indicate outliers that lie beyond 3-times the inter quartile range. We analyzed skill transfer by comparing the differences of the weeks 1 and 2; for the retention part, we evaluated differences between day 4 and day 3 of week 2. The retention *p*-values are Bonferroni-corrected for multiple testing, because the long-term retention test was done with a subgroup of this data set

We calculated the differences in kinematic assessments, the BBT, and the Melbourne for week 1 and compared these to the differences for week 2 to determine the transfer (Fig. 3 and Table 2).

**Table 2:** Acquisition, transfer, and retention values

	Week 1		Week 2			Transfer Δweek 2 – Δweek 1		Retention Week 2 day 4 - day 3	
	Day 1	Day 3	Day 1	Day 3	Day 4	*p*-value	Effect size	*p*-value	Effect size
Time [s]	25.55 ± 5.56	24.64 ± 7.37	22.09 ± 4.48	20.05 ± 4.93	18.86 ± 3.13	0.433	d = 0.32	0.337	d = 0.29
Path-ratio [m/m]	1.81 ± 0.48	1.84 ± 0.50	1.70 ± 0.31	1.50 ± 0.26	1.47 ± 0.22	0.266	r_ES_ = 0.24	0.880	d = 0.10
BBT [blocks/min]	36.59 ± 15.15	37.05 ± 14.01	36.50 ± 13.99	40.46 ± 14.63	39.82 ± 15.24	0.008	d = 1.59	0.559	d = 0.04
Melbourne [%]	90.34 ± 9.78	89.96 ± 9.81	90.34 ± 9.28	92.24 ± 8.13	91.86 ± 9.20	0.063	r_ES_ = 0.40	0.996	r_ES_ = 0.14

Displayed are the means ± standard deviations for all measurement time points of week 1 and 2. Skill transfer analyzes the difference between week 1 and 2, whereas retention looks at the difference of day 3 and 4 of week 2

*Abbreviations*: *d* Cohan’s d, *r*_*ES*_ non-parametric effect size, *BBT* Box and Block Test, *Melbourne* Melbourne Assessment for Unilateral Upper Limb Function

For the path-ratio, a Wilcoxon signed rank test revealed no significant difference from week 1 (mean = 0.03, SD = 0.19) to week 2 (mean = −0.20, SD = 0.40; *T* = 20.50, *p* = 0.266) and the effect size was small (r_ES_ = 0.24). Also, the parameter time of the kinematic assessments did not improve significantly (mean difference of week 1 = −0.91 s, SD = 3.69 s, versus mean difference of week 2 = −2.05 s, SD = 3.44 s; *t*(10) = −0.82, *p* = 0.433). The effect size was small (d = 0.32).

For the BBT, the difference of transferred blocks per minute was significantly greater for week 2 (mean = 3.95, SD = 2.55) than for week 1 (mean = 0.45, SD = 1.80; *t*(10) = 3.33, *p* = 0.008). The effect size was well above the threshold to be considered large (d = 1.59). The differences in percentage score of the Melbourne Assessment of week 1 (mean = −0.38, SD = 5.17) did not significantly differ from those of week 2 (mean = 1.89, SD = 3.89; *T* = 25.00, *p* = 0.063), but the effect size could be considered medium (r_ES_ = 0.40).

### Retention

As shown in Fig. 2, the game scores at the end of the training phase and at retention (i.e. one day later) did not differ significantly [*t*(10) = −0.11, *p* = 1.00, Bonferroni corrected]. The effect size was below the criterion to be considered small (d = 0.01).

For the BBT (Fig. 3 and Table 2), the difference between day 3 and 4 was not significant [*t*(10) = −1.14, *p* = 0.559] and the effect size was also below small (d = 0.04).

We were able to evaluate the 8-day retention in 6 of 11 participants. The BBT improved during week 2 from 39.5 ± 11.1 blocks/min (mean ± SD) on day 1 to 43.5 ± 10.6 on day 3. On day 4, the BBT amounted to 43.3 ± 12.2 and this did not differ from the BBT score one week later (43.0 ± 12.1 blocks/min). For the BBT, there was no significant difference between day 3 of week 2 and 8 days later [t(5) = −0.55, *p* = 1.00]. Again, the effect size was below the threshold to be considered small (d = 0.04).

As the kinematic assessments and the Melbourne Assessment did not improve (Fig. 3 and Table 2), we did not investigate the retention of these assessments.

### Possible confounder: Number of therapies between assessments

When comparing the number of therapies of week 1 to week 2, we found that the participants had significantly more occupational therapy sessions during week 2 (median = 1, mean = 0.82, SD = 0.75) than during week 1 (median = 0, mean = 0.45, SD = 0.69; *T* = 10.00, *p* = 0.046). We did not observe such a difference for any other therapy. The number of occupational therapy sessions did not correlate significantly with any of the outcome measures. The Spearman correlation coefficients varied between −0.24 and 0.12.

## Discussion

This study assessed whether weight-supported playful training in a virtual environment could result in the acquisition, transfer, and retention of upper limb skills in children with CP. The main results were that 70 min of pure playtime resulted in significant improvements of the exergame score and the Box and Block Test, indicating skill acquisition and transfer. While two kinematic assessments (path-ratio and time needed to perform the task) and the Melbourne Assessment did not improve significantly, a medium effect size for the Melbourne Assessment was found. Finally, 24 h retention occurred for the game score and the BBT.

### Skill acquisition

One important factor to infer whether motor learning has occurred is the improvement of the task during acquisition. Indeed, we could show that 3 days of 20 to 30 min of intense exergaming was sufficient to improve the exergame scores. This dosage is relatively low when compared to other studies. For example, Geerdink et al. [30] evaluated motor learning curves based on BBT results for children aged 2.5 to 8 years with CP, who had received 6 weeks (54 h) of constrained induced movement therapy followed by 2 weeks (18 h) of bimanual training. BBT results improved during the first 6 weeks, after which they showed a decline. When referring to studies who also used novel technologies for the upper extremity, Krebs [9] observed improvements in an accurate pointing task with the InMotion2 device after 16 1 h training sessions. Participants performed 640 to 960 pointing movements per session and showed improvements in various outcomes. In the study by Gilliaux et al. [13], 8 children with CP completed 3 conventional therapy sessions and 2 robot-assisted sessions per week over 8 weeks. During each robotic session with the REAPlan robot, a distal end-effector robot that allows for displacements of the upper limb in the horizontal plane, the participants performed on average about 744 movements.

These dosages are clearly beyond what we achieved in this study. While the overall duration of exergaming amounted to 70 min, the ARMEO® Spring device did not report outcomes on the amounts of target movements performed when playing the Moorhuhn game. We therefore estimated the number of point-to-point and grasping movements from the game scores. In a worst-case calculation, merely dividing the overall average game score (279.8 ± 45.5) by the maximal game score per target (25 points) would result in an average of about 11 point-to-point movements per trial (i.e. extrapolated to the study: 770 pointing movements for the 3-day intervention). However, based on the feedback from a reviewer, we performed an additional experiment. Two participants with CP who would have fitted the inclusion criteria of the original study (both had an ataxic CP; 1 boy, 1 girl; 16 and 14 years, MACS II and I, respectively) were familiarized with the game (first a demonstration and then 5 one-minute test trials). Then, each participant played 10 one-minute trials of Moorhuhn, while we videotaped the game. Two raters scored afterward independently from each other the video recordings. Participant 1 achieved an average exergame score of 294 ± 23 points and performed on average 47.2 ± 13.4 (rater 1) or 45.7 ± 11.4 (rater 2) point-to-point grasping movements. Participant 2 scored on average 344 ± 67 points. The counted point-to-point grasping movements of participant 2 amounted to 40.4 ± 5.8 (rater 1) or 40.1 ± 5.5 (rater 2). We, therefore, assume that 43 (grand average) point-to-point grasping movements per minute game playing would be a more realistic estimation, which would lead to around 70 × 43 = 3010 point-to-point grasping movements for the 3-day intervention in total.

The number of repetitions per session in our study appears similar to those from Krebs and colleagues [9] or Gilliaux et al. [13], but we had considerably less sessions. Naturally, the total amount of movement repetition is a deciding factor for the success of an intervention and more therapy sessions may have led to results that are more distinct.

Certainly, skill acquisition plays an important role in the explanation of our findings. However, in our endeavor to select an engaging exergame which encourages performing as many movement repetitions as possible in a short time period, we also selected a game where decision-making could contribute to the observed improvements. A player can influence the score not only by shooting more birds but also by selecting targets of greater value. This could mean that attributing gains to skill acquisition alone would be a misinterpretation, however, hitting birds worth more points could also indicate improved skilled performance, as these birds were smaller and required more-precise aiming movements.

### Transfer

The combination of the ARMEO® Spring device with the Moorhuhn exergame resulted in practicing to move and position the arm and hand quickly and accurately in a virtual environment and timely grasp the joystick to shoot at chickens. We were rather surprised that in a fairly similar transfer task, namely the kinematic assessments, we found no significant improvements in path-ratio or time needed to perform the task. The lack of significance was not just due to a small statistical power, because also the effect sizes were only small. In contrast, other studies detected changes in kinematic parameters for adult stroke patients [31–33] as well as children affected by CP [9, 13] when training with assistive robots. Besides the differences in dosage (total number of movements, as discussed in the previous paragraph) between these studies and the current one, the kinematic assessment might also play a role. Speed and accuracy are interlocked (speed-accuracy tradeoff function), meaning more errors occur when a movement is performed faster and conversely speed decreases in order to increase accuracy [14]. To indicate true skill acquisition, there has to be a change in the speed-accuracy tradeoff function [34], which we could not test for in our study. Furthermore, we tested children who were able to perform the BBT and therefore had only mild to moderate impairment. As a result, they were already relatively good in the kinematic assessment and, indeed, in some participants we could observe a certain ceiling effect, which might have contributed to the lack of significant improvement we found in this study.

As a quantitative outcome measure for transfer of skill, the BBT was chosen. It is an easily explained, time-efficient assessment, which shows no ceiling effect and evaluates the most essential hand functions, i.e. grasp, hold, transfer, and release [30]. We found a significant difference (3.5 blocks/min) between the weeks in favor of the intervention week accompanied by a huge effect size. Other studies found results of similar magnitude for the BBT after robot-assisted therapy for adults with stroke (3.9 blocks/min) [35] as well as children affected by CP (3.6 blocks/min) [13]. How is it possible that, with similar intensities but only a fraction of the training volume, we found results in the same range? Firstly, in this study the children trained in a device supporting 3-dimensional movements (despite that the exergame required only 2 dimensional movements) as opposed to training in a fix plane. This may have had a positive influence, since the BBT also requires vertical, not only planar movement. Secondly, as mentioned above, the BBT evaluates other hand functions (e.g. grasping) besides point-to-point movements. When playing “Moorhuhn” the grasping and releasing part of the assessment were addressed as well. This may have led to relatively large changes in a short amount of time. Thirdly, we included also mildly impaired patients in our study, which was an advantage because the BBT does not show a ceiling effect. Despite that the changes measured in this study might not exceed the threshold to be considered clinically relevant [13, 36], one must not forget that the participants attained them in only 3 20–30 min long intervention sessions.

We also applied the Melbourne Assessment. Its ordinal scale takes into account qualitative aspects of movement. The Melbourne Assessment is valid and reliable, even if the items are looked at separately [26, 37]. The Melbourne Assessment improved with a medium effect size accompanied by a trend towards significance. Seemingly, the sample size was too small to detect a statistically significant improvement. But particularly for this assessment, the ceiling effect might have influenced the results considerably. As displayed in Table 2, the participants were already very good at onset of the study and could improve on average less than 1 point. Schneiberg et al. [38] reported the Melbourne Assessment as being less sensitive than kinematic measures, since they too found no significant differences. This makes sense, because the Melbourne Assessment divides movement quality in rough ordinal categories and thus cannot detect fine improvements in the smoothness of movement for example.

Overall, we can say that the children were able to transfer the acquired skill to one clinical test. The discrepancy between the findings of the BBT and the Melbourne Assessment may be due to either sensitivity issues or the fact that the improvements need to be attributed to functional compensation rather than recovery of impairment. The BBT is a functional test, indicating recovery of function and not considering quality of movement, thus it does not discriminate between recovery of impairment and functional compensation [14].

### Retention

Finally, we evaluated whether children were able to maintain the gains for at least 24 h and thus cover another important part of motor learning, retention. As we found no significant improvements in the kinematic assessment and the Melbourne Assessment, retention was limited to the exergame score and the BBT results. Indeed, for both measures we could show that the gains retained for at least 24 h.

We knew from clinical experience that it was hard to plan a retention measurement 8 days after the final training, due to the patient’s discharge. While we could perform these measurements only in half of the participants, the results showed that the improvements in BBT were maintained up to 8 days after finishing three days of exergaming. Of course, in our study, participants received conventional therapies during this period, which might have prevented a potential deterioration.

### Therapies between assessments

The results of the comparisons between the therapy sessions need to be analyzed, because the study is built around the premise that the performance differences can be attributed to the ARMEO® Spring intervention and not the ongoing therapies of the rehabilitation schedule. As previously shown, significant differences favoring week 2 were found for occupational therapy. It should not be forgotten that the differences were, absolutely speaking, very small (from on average less than half an occupational session to 0.8 occupational sessions). In addition, the exact content of the occupational therapies is not known and may not solely include working on hand and arm functioning. The correlations were indeed small and not significant indicating that the differences in number of occupational therapy sessions did not correlate with the observed improvements in upper extremity skills.

### Methodological considerations

Throughout the discussion, we addressed certain methodological considerations in their context, one of them being the small number of participants. Even though the sample size was small, we were still able to find significant differences with effect sizes indicating that they were also meaningful. Another point of emphasis was the impairment of the children being mild to moderate. Therefore, we cannot generalize our findings to children who are more severely impaired. Future studies with larger numbers of participants, including severely affected children, might be able to determine which subgroups can profit the most from assisted training.

Training volume was a further limitation of this study. For practical reasons, based on length of stay in our rehabilitation center, we had to choose a very short protocol. By measuring baseline outcomes to control for a potential effect of the conventional rehabilitation treatments, we had even less time to train with the ARMEO® Spring. As mentioned, there are studies on this topic with much longer training periods [9, 13] and retention tests indicating that improvements gained over a longer period of time are retained at least a month [9]. Further research in this area might be interested in determining how much training is necessary to induce clinically relevant improvements or when children reach learning plateaus by regularly evaluating the gains throughout the intervention [30]. Also, looking at the progression of outcome measures following an intervention may provide evidence as to how often such rehabilitation blocks should take place to yield the best possible results for patients.

Another problem we encountered, even during our very short intervention, was cognition and motivation. We included children only when they seemed cognitively fit and compliant (we evaluated WeeFIM cognition scores and asked responsible therapists). ID4 was not compliant, and we excluded ID5 at his request because he found the game boring. Two other participants seemed reluctant to play after the first training day but continued. Because our goal was to demonstrate a learning curve with the game score we decided to play only Moorhuhn for three consecutive days, however, to keep the children motivated over longer periods of training, it may be advantageous to add a variety of games, adjustable difficulty levels or even other tasks to the protocol. As a side benefit this contextual interference effect may lead to better retention of the learned tasks [14].

Assessments were not performed by a blinded assessor. While some assessments were performed automatically in the ARMEO® Spring, we recommend for future studies a blinded assessment, especially for the clinical assessments, to reduce the risk of bias.

Last but not least, the fact that the kinematic assessments were performed with assistance-as-needed has to be addressed. We tried to give the children as little support as possible but since the movements were aided that may have led to less pronounced differences, making it more difficult to detect improvements. Then again, the ARMEO® Spring may have limited the range of motion, especially the flexion of the shoulder with an extended elbow. One child displayed difficulty in reaching certain targets due to limited mobility. In general, this may have led to patients changing their movement patterns and flexing their elbow (reduced lever) while raising their arm in order to reach targets that are more elevated.

Furthermore, there is a discrepancy between the mode of training (workspace was calibrated for each patient individually) and the kinematic assessment ‘vertical catch 2D’ (fixed workspace). This might have made the transition from the game to the assessment and back more difficult since identical movements of the limb in real life lead to slightly different movements of the cursor in the assessment and the game environment.

## Conclusion

Seventy minutes of “Moorhuhn” playing resulted in a significant improvement of the exergame performance and the Box and Block Test, indicating skill acquisition and transfer. These gains were retained for 24 h (or even 8 days). It was unlikely that the differences were caused by conventional therapeutic interventions. We therefore infer that motor learning occurred when these children with CP trained with weight-support in a playful, virtual environment.

## Abbreviations

2D: 2 dimensional
3D: 3 dimensional
BBT: Box and Block Test
CI: Confidence interval
Cog: Cognition domain of the WeeFIM
CP: Cerebral Palsy
DF: Degrees of freedom
GMFCS: Gross Motor Function Classification System
ID: Identification number
MACS: Manual Ability Classification System
Mob: Mobility domain of the WeeFIM
R^2^: Explained variance
S-C: Self-care domain of the WeeFIM
SD: Standard Deviation
SEM: Standard error of the mean
WeeFIM: Functional Independence Measure for children
